# Mechanisms of α-Synuclein Induced Synaptopathy in Parkinson's Disease

**DOI:** 10.3389/fnins.2018.00080

**Published:** 2018-02-19

**Authors:** Jessika C. Bridi, Frank Hirth

**Affiliations:** King's College London, Department of Basic and Clinical Neuroscience, Maurice Wohl Clinical Neuroscience Institute, Institute of Psychiatry, Psychology and Neuroscience, London, United Kingdom

**Keywords:** Parkinson's disease, synapse, SNARE complex, active zone, dopamine, α-synuclein, synaptopathy, neurodegeneration

## Abstract

Parkinson's disease (PD) is characterized by intracellular inclusions of aggregated and misfolded α-Synuclein (α-Syn), and the loss of dopaminergic (DA) neurons in the brain. The resulting motor abnormalities mark the progression of PD, while non-motor symptoms can already be identified during early, prodromal stages of disease. Recent studies provide evidence that during this early prodromal phase, synaptic and axonal abnormalities occur before the degenerative loss of neuronal cell bodies. These early phenotypes can be attributed to synaptic accumulation of toxic α-Syn. Under physiological conditions, α-Syn functions in its native conformation as a soluble monomer. However, PD patient brains are characterized by intracellular inclusions of insoluble fibrils. Yet, oligomers and protofibrils of α-Syn have been identified to be the most toxic species, with their accumulation at presynaptic terminals affecting several steps of neurotransmitter release. First, high levels of α-Syn alter the size of synaptic vesicle pools and impair their trafficking. Second, α-Syn overexpression can either misregulate or redistribute proteins of the presynaptic SNARE complex. This leads to deficient tethering, docking, priming and fusion of synaptic vesicles at the active zone (AZ). Third, α-Syn inclusions are found within the presynaptic AZ, accompanied by a decrease in AZ protein levels. Furthermore, α-Syn overexpression reduces the endocytic retrieval of synaptic vesicle membranes during vesicle recycling. These presynaptic alterations mediated by accumulation of α-Syn, together impair neurotransmitter exocytosis and neuronal communication. Although α-Syn is expressed throughout the brain and enriched at presynaptic terminals, DA neurons are the most vulnerable in PD, likely because α-Syn directly regulates dopamine levels. Indeed, evidence suggests that α-Syn is a negative modulator of dopamine by inhibiting enzymes responsible for its synthesis. In addition, α-Syn is able to interact with and reduce the activity of VMAT2 and DAT. The resulting dysregulation of dopamine levels directly contributes to the formation of toxic α-Syn oligomers. Together these data suggest a vicious cycle of accumulating α-Syn and deregulated dopamine that triggers synaptic dysfunction and impaired neuronal communication, ultimately causing synaptopathy and progressive neurodegeneration in Parkinson's disease.

## Introduction

**Figure 1:**
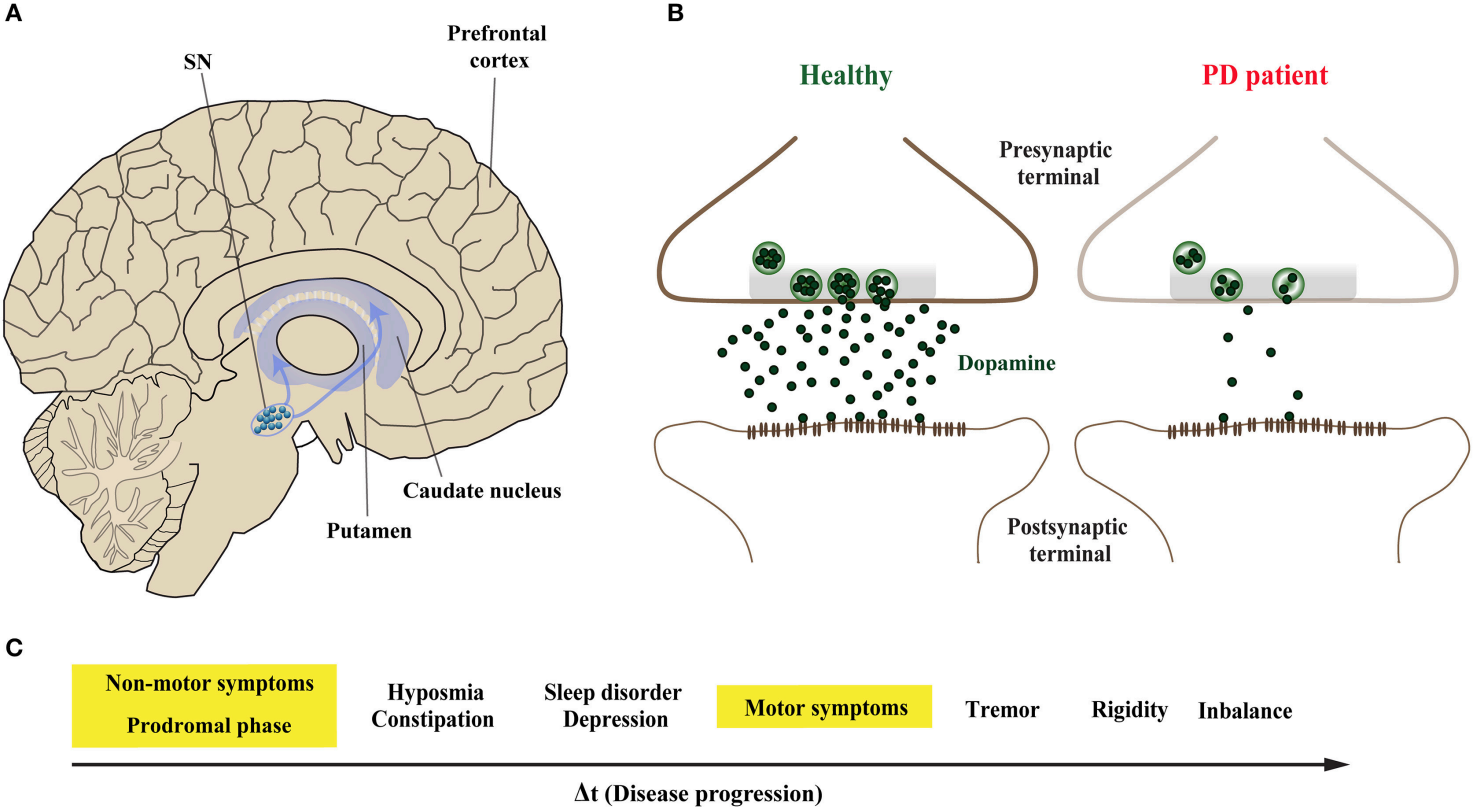
Midbrain dopaminergic neurons are specifically vulnerable in Parkinson's disease. **(A)** The predominant symptoms of Parkinson's disease (PD) are caused by loss of dopaminergic (DA) neurons in the substantia nigra (SN). According to the dying-back hypothesis, the degeneration of DA neurons is preceded by dysfunction and in turn degeneration of the nigrostriatal pathway, which innervates the caudate nucleus and the putamen that together form the striatum. **(B)** Compared to healthy controls (left), nigrostriatal degeneration results in the depletion and ultimate loss of the neurotransmitter dopamine on synaptic terminals of striatal neurons (right). **(C)** The resulting motor symptoms, among others, are usually diagnosed when approximately 30–60% of striatal DA neurons are already lost. However, PD patients can experience non-motors symptoms 20 years before the onset of motor abnormalities in the so-called prodromal phase; these include olfactory dysfunction, sleep disturbances and depression.

Parkinson's disease (PD) is the second most common neurodegenerative disorder after Alzheimer's disease (AD) (Kalia and Lang, 2015). The pathological hallmarks of PD are intracellular proteinaceous inclusions called Lewy bodies (LB) and Lewy neurites (LN) that are predominantly formed of misfolded and aggregated forms of the presynaptic protein α-Synuclein (α-Syn), and the loss of dopaminergic (DA) neurons in the substantia nigra (SN) (Spillantini et al., 1997; Lang and Lozano, 1998a,b). Loss of DA neurons in the SN leads to marked decrease of dopamine levels in synaptic terminals of the dorsal striatum (Figures 1A,B), ultimately leading to a loss of the nigrostriatal pathway (Cheng et al., 2010). The reduction of striatal dopamine triggers a range of motor symptoms including bradykinesia, uncontrollable tremor at rest, postural impairment, and rigidity which together characterize PD as a movement disorder.

The onset of PD, however, is considered to commence at least 20 years prior to detectable motor abnormalities, when a variety of non-motor symptoms can be observed (Hawkes et al., 2010; Kalia and Lang, 2015; Mahlknecht et al., 2015). This period is referred to as the prodromal phase where patients experience a range of non-motor symptoms including constipation, olfactory dysfunction (hyposmia), sleep disturbance, obesity and depression (Figure 1C; Hawkes et al., 2010; Kalia and Lang, 2015; Mahlknecht et al., 2015). During the prodromal phase of PD and PD-related disorders, which precedes degenerative cell loss, the expression levels of a range of proteins involved in synaptic transmission are altered in the prefrontal and cingulate cortex, and SN (Dijkstra et al., 2015; Bereczki et al., 2016; Table 1), suggesting that both non-motor and motor symptoms are caused by impaired synaptic communication. This data indicates that neurodegeneration in PD is a dying back-like phenomenon which starts at synaptic terminals in the striatum and progresses along the nigrostriatal pathway, ultimately affecting homeostasis and survival of DA cell bodies in the SN (Hornykiewicz, 1998; Calo et al., 2016; Caminiti et al., 2017). It is due to these early-onset synaptic alterations observed prior to DA neuron loss, PD has also been classified as a synaptopathy (Brose et al., 2010; Schirinzi et al., 2016).

**Table 1:** Alterations of presynaptic proteins seen with α-Syn related proteinopathy in PD and DLB patients and rodent animal models.

**Protein**	**Modification**	**Species**	**Assay system**	**References**
Synapsin 1	Down	Human	Post-mortem tissue from DLB patients	Scott et al., 2010
		Rat	BAC rat model overexpressing the full-length human SNCA	Kohl et al., 2016
		Mouse	Hippocampal neurons overexpressing human WT α-Syn:GFP	Scott et al., 2010
		Mouse	Overexpression of human WT α-Syn	Nemani et al., 2010
Synapsin 2	Down	Human	Post-mortem tissue from PD patients (Braak stages 1–2)	Dijkstra et al., 2015
		Mouse	Overexpression of human WT α-Syn	Nemani et al., 2010
		Mouse	Primary neuronal culture treated with α-Syn pre-formed fibrils	Volpicelli-Daley et al., 2011
Synapsin 3	Down	Human	iPSCs from patients harboring A53T mutation	Kouroupi et al., 2017
	Up	Human	Post-mortem tissue from PD patients (Braak stages 1–2)	Dijkstra et al., 2015
Synaptophysin	Down	Human	Post-mortem tissue from DLB patients	Kramer and Schulz-Schaeffer, 2007
		Mouse	Overexpression of human α-Syn under the mThy1 promoter	Games et al., 2014
	Up	Human	Post-mortem tissue from PD patients (Braak stages 1–2)	Dijkstra et al., 2015
CSPα	Down	Mouse	Primary neuronal culture treated with α-Syn pre-formed fibrils	Volpicelli-Daley et al., 2011
SV glycoprotein 2	Down	Human	iPSCs from patients harboring A53T mutation	Kouroupi et al., 2017
Rab3A/Rab3	Down	Human	iPSCs from patients harboring A53T mutation	Kouroupi et al., 2017
		Human	Post-mortem tissue from PDD and DLB patients	Bereczki et al., 2016
		Rat	Nigral injection of AAV2-A53T-α-Syn	Chung et al., 2009
		Mouse	Overexpression of human WT α-Syn	Nemani et al., 2010
VAMP-2	Down	Mouse	Hippocampal neurons overexpressing human WT α-Syn:GFP	Scott et al., 2010
		Mouse	Primary neuronal culture treated with α-Syn pre-formed fibrils	Volpicelli-Daley et al., 2011
	Misloc.	Human	Post-mortem tissue from PD patients (striatum-putamen and external globus pallidus)	Garcia-Reitböck et al., 2010
	Up	Mouse	Truncated human α-Syn	Garcia-Reitböck et al., 2010
		Mouse	Overexpression of human WT α-Syn	Nemani et al., 2010
SNAP-25	Down	Human	Post-mortem tissue from PDD and DLB patients	Bereczki et al., 2016
		Mouse	Primary neuronal culture treated with α-Syn pre-formed fibrils	Volpicelli-Daley et al., 2011
	Misloc.	Human	Post-mortem tissue from PD patients (striatum-putamen and external globus pallidus)	Garcia-Reitböck et al., 2010
		Mouse	Truncated human α-Syn	Garcia-Reitböck et al., 2010
	Up	Mouse	Overexpression of human WT α-Syn	Nemani et al., 2010
Syntaxin-1/Syntaxin	Down	Human	Post-mortem tissue from DLB patients	Kramer and Schulz-Schaeffer, 2007
		Rat	Nigral injection of AAV2-A53T-α-Syn	Chung et al., 2009
		Mouse	Overexpression of human WT α-Syn	Nemani et al., 2010
	Misloc.	Human	Post-mortem tissue from PD patients (striatum-putamen and external globus pallidus)	Garcia-Reitböck et al., 2010
		Mouse	Truncated human α-Syn	Garcia-Reitböck et al., 2010
Synaptotagmin 1	Up	Mouse	Overexpression of human WT α-Syn	Nemani et al., 2010
Synaptotagmin 2	Up	Human	Post-mortem tissue from PD patients (Braak stages 1–2)	Dijkstra et al., 2015
Complexin 1	Down	Mouse	Overexpression of human WT α-Syn	Nemani et al., 2010
Complexin 2	Down	Human	Post-mortem tissue from PD patients (Braak stages 1–2)	Dijkstra et al., 2015
		Mouse	Overexpression of human WT α-Syn	Nemani et al., 2010
Synphilin 1	Down	Human	Post-mortem tissue from PD patients (Braak stages 1–2)	Dijkstra et al., 2015
Munc18-1	Up	Mouse	Overexpression of human WT α-Syn	Nemani et al., 2010
Munc13-1	Down	Mouse	Overexpression of human WT α-Syn	Nemani et al., 2010
Piccolo	Down	Mouse	Hippocampal neurons overexpressing human WT α-Syn:GFP	Scott et al., 2010
RIM3	Down	Rat	BAC rat model overexpressing the full-length human SNCA	Kohl et al., 2016
Amphiphysin	Down	Mouse	Hippocampal neurons overexpressing human WT α-Syn:GFP	Scott et al., 2010

The majority of PD cases are sporadic with unknown cause. However, familial cases with autosomal dominant or recessive Mendelian inheritance have revealed fundamental insights into pathogenic mechanisms underlying PD (Keane et al., 2011; Massano and Bhatia, 2012; Kalia and Lang, 2015). The most commonly identified genetic mutations linked to heritable PD were found in the genes *SNCA* and *LRRK2*, responsible for an autosomal-dominant forms of PD, and in *Parkin, PINK1, DJ-1*, and *ATP13A2* which account for PD with autosomal recessive mode of inheritance (Klein and Westenberger, 2012; Ferreira and Massano, 2017). PD-related mutations in *PINK1* and *Parkin* and the functional interaction of the two proteins led to the identification of mitochondrial dysfunction as one of the major pathogenic pathways underlying PD (reviewed in Exner et al., 2012; Pickrell and Youle, 2015). Additionally, PD-related mutations in *Glucocerebrosidase* (*GBA*), *SCARB2* and *ATP13A2* established lysosomal storage dysfunction as a second pathogenic pathway that also contributes to PD etiology (reviewed in Hardy, 2010; Gan-Or et al., 2015).

However, among the identified PD-related genes, *SNCA* encoding the presynaptic protein α-Syn remains the most potent culprit underlying PD. Accumulated α-Syn is the main component of LB, and together with genome-wide association studies it has been shown to have central pathogenic role in both familial and sporadic PD (Satake et al., 2009; Simón-Sánchez et al., 2009). Yet despite recent progress, the pathogenic mechanisms underlying α-Syn related PD are only starting to emerge. Here we provide a focussed review emphasizing current knowledge on synaptic function and the various mechanisms of α-Syn induced synaptopathy, and its role in the early stages of PD progression.

## Accumulation of α-Synuclein: the pathological hallmark of PD

**Figure 2:**
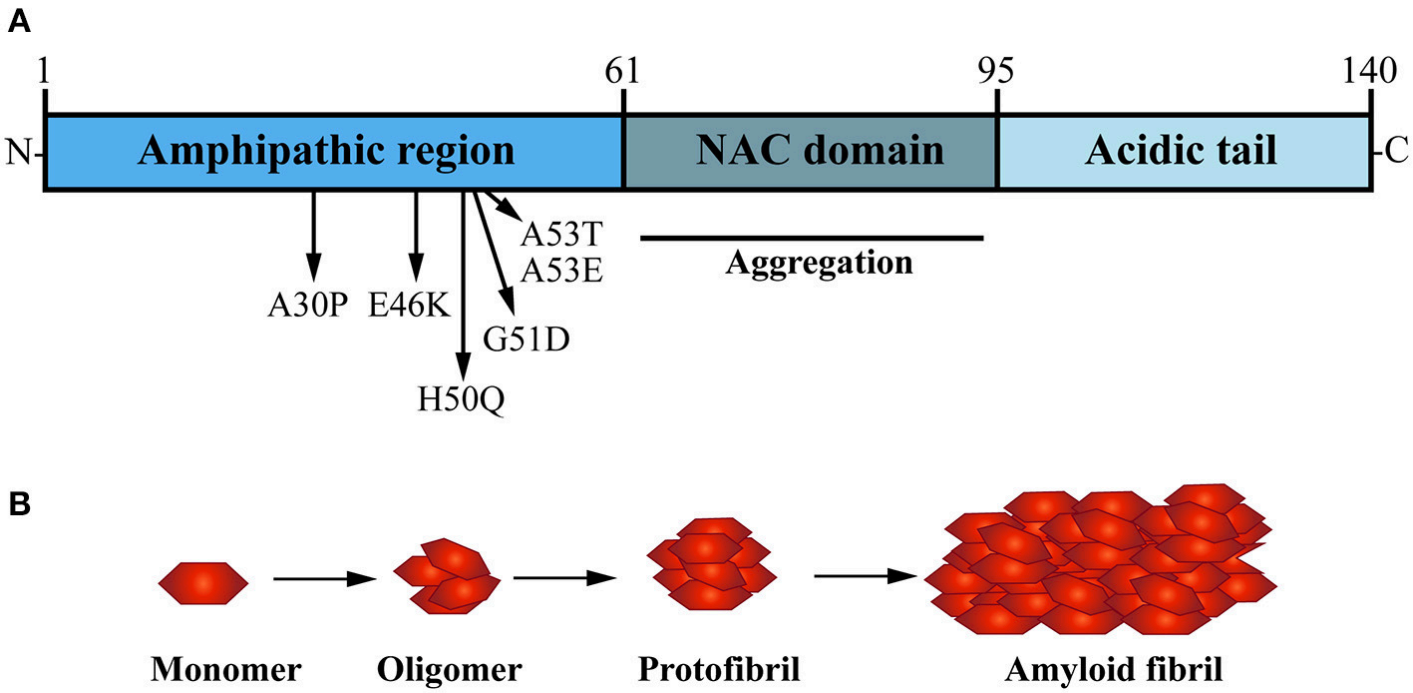
The presynaptic protein α-Synuclein is a pathological seed for Parkinson's disease formation. **(A)** α-Synuclein (α-Syn) is a small soluble cytoplasmic protein of 140 amino acid encoded by the SNCA gene; its main protein domains comprise an N-terminal amphipathic region, a non-amyloid-β component (NAC) domain and a C-terminal acidic tail. Several dominant inherited missense mutations have been identified in the amphipathic region causing early-onset PD, whereas the NAC domain has been implicated in α-Syn aggregation. **(B)** Under normal physiological conditions, α-Syn monomers function in a dynamic equilibrium between a soluble and a membrane-bound state. Under cellular stress and in disease-relation conditions, α-Syn monomers can interact leading to oligomers that buffer the formation of protofibrils, ultimately causing the formation of amyloid-β sheet fibrils that aggregate into Lewy bodies (LB).

α-Syn is a small soluble cytoplasmic protein of 140 amino acid; its main protein domains comprise an amphipathic region, non-amyloid-β component (NAC) domain and an acidic tail (Figure 2A; Maries et al., 2003; Venda et al., 2010). α-Syn together with β-Syn and γ-Syn belong to the synuclein protein family (Goedert, 2001; Marques and Outeiro, 2012). The three Synuclein proteins are 55–62% identical in sequence and have similar domain organization (Goedert, 2001); all three are highly expressed in the human brain (Goedert, 2001). Additionally, α-Syn and β-Syn share the same subcellular distribution at presynaptic terminals in neurons (Jakes et al., 1994; Goedert, 2001). However, α-Syn is the only protein of the synuclein family to be found in LB and to be implicated in PD pathogenesis (Goedert et al., 2017).

Several dominant-inherited single point mutations in *SNCA* gene have been identified in families that develop early onset PD (Polymeropoulos, 1997; Krüger et al., 1998; Zarranz et al., 2004; Appel-Cresswell et al., 2013; Lesage et al., 2013; Pasanen et al., 2014). Subsequent studies identified multiplications of the *SNCA* gene locus (Singleton, 2003; Farrer et al., 2004) and *SNCA* polymorphisms which are associated with high risk of developing the disease (Satake et al., 2009; Simón-Sánchez et al., 2009). In line with dosage-related α-Syn pathology, higher doses of α-Syn observed in patients with duplication and triplication of the *SNCA* gene locus directly correlate with cognitive decline, motor and non-motor symptoms and severity of neurodegenerative phenotypes (Venda et al., 2010). This data is strong evidence that dysfunction and accumulation of α-Syn plays a key role in both familial and sporadic forms of PD. In addition to LB inclusions, α-Syn has been shown to accumulate in axons (Braak et al., 1999) and in the presynaptic terminal (Kramer and Schulz-Schaeffer, 2007), suggesting these pathogenic accumulations are a key trigger of onset and formation of PD-related synaptopathy (Stefanis, 2012; Schirinzi et al., 2016) (Box 1).

Box 1 **α-Syn-mediated synaptopathy - open questions and future directions.**

Progress over the past two decades revealed compelling evidence that one of the major sites of α-Syn-mediated toxicity are presynaptic terminals. Once initiated, the resulting neuronal dysfunction can lead to both cell intrinsic and cell-extrinsic toxicity. Cell-intrinsic mechanisms cause axonal degeneration which progresses toward the cell body, eventually leading to dying-back like neurodegeneration. Cell-extrinsic mechanisms cause transmission of α-Syn oligomers along synaptic connections and the ultimate progression of synucleopathy throughout the patient brain. Neither of these mechanisms are fully understood, nor how α-Syn oligomerizes in the first place, thus precluding the development of efficient therapeutic treatments. To progress toward therapy, several open questions need to be addressed.

***Conformational changes of α-Syn***

α-Syn accumulates in synapses and axons and forms neuronal inclusions. What triggers α-Syn self-assembly and in turn the formation of toxic species of misfolded α-Syn? How do different types of oligomers serve as template for fibril formation of α-Syn? There is evidence that the formation of fibrils, rather than toxic species themselves, mediates α-Syn toxicity (Lashuel et al., 2013). Which aspect of this process is so pathogenic to neurons? It also remains to be shown whether α-Syn may form various types of oligomeric states with different toxicity. To address these questions in a systematic way, first and foremost reliable markers are required to monitor, both *in vitro* and *in vivo*, the different and progressive conformational changes of α-Syn and their impact on toxicity.

α-Syn acts as a chaperone for the presynaptic SNARE complex. It controls assembly, maintenance and distribution of this highly reactive complex of proteins. Can this environment be responsible for triggering the pathologic cascade to form α-Syn oligomers and fibrils? The increased stability and longer half-life of mutant forms of α-Syn, such as A30P and A53T, increase their potential of aggregation (Bennett et al., 1999; Cuervo et al., 2004). In addition, failure of proteasomal degradation and autophagy in PD have been linked to high levels of α-Syn. Together, these factors can favor the accumulation of pathologic α-Syn. Small molecule and compound screens together with *in vivo* genetic screens in animal models are necessary to identify mechanisms and molecules that can abrogate, hamper or at least delay misfolding of α-Syn, and thus progression of disease.

***Accumulation of α-Syn and synaptic defects***

What is the threshold concentration of toxic α-Syn species, such as oligomers and fibrils, to trigger biochemical and cellular alteration that lead to neuronal dysfunction? Oligomeric forms of α-Syn are considered the most toxic species at the synapses (Ingelsson, 2016) and their structure appears different between mutant forms and wild type α-Syn (Tosatto et al., 2015). This suggests the severity of pathology may not only relate to the abundance and formation of oligomers but also to their structural and functional properties. It remains to be shown whether distinct oligomeric α-Syn species, and their way of formation, exert differential toxic mechanisms that specifically impair the presynaptic terminal and neurotransmission.

Do oligomeric and fibrils of α-Syn cause a dominant negative effect in synaptic function? Can α-Syn oligomers, for instance, reduce the activity of its synaptic partners and neuronal signaling pathways? The effect of different lengths and concentrations of α-Syn oligomers on SNARE-mediated membrane fusion and docking are one way to measure toxicity, as previously shown *in vitro* (Choi et al., 2013; Lai et al., 2014). *In vitro* protein-reconstituted systems are necessary to test the effects of different toxic α-Syn species on fast Ca^2+^-triggered neurotransmitter release and their *in vivo* toxicity in synaptic transmission.

***α-Syn transmission and transcellular progression of disease***

Several studies using animal models have given strength to Braak's hypothesis for α-Syn propagation within interconnected brain regions (reviewed in Oueslati et al., 2014). However, the mechanisms underlying α-Syn transmission remain poorly understood. Which α-Syn toxic species are “seeding” for trans-synaptic transmission? What is the minimum concentration of toxic α-Syn required to trigger transmission? Can extracellular α-Syn and exosomes be efficiently cleared by pharmacological or gene therapy approaches without affecting the intracellular endogenous α-Syn? To address these questions, *in vivo* systems to allow monitoring of α-Syn transmission are required. For example, *Drosophila melanogaster* has been successfully used to study transcellular spreading of human huntingtin (Htt) (Babcock and Ganetzky, 2015). This model system has opened avenues for unbiased whole genome screening of cellular mechanisms and pharmacological interventions of Htt transmission. α-Syn transmissibility could also be studied in brain organoids derived from induced pluripotent stem cells (iPSCs) of healthy individuals and patients harboring α-Syn mutations. Although an *in vitro* culture, organoids resemble the *in vivo* architecture, functionality, and genetic signature of original tissues (Dutta et al., 2017). Moreover, organoids develop neuronal networks that can be modulated by sensory stimulation (Quadrato et al., 2017). Hence organoids can be used to study α-Syn propagation along interconnected neuronal networks.

In the healthy brain and central nervous system (CNS), wild type α-Syn is available in its native conformation as soluble monomers. These are thought to mediate its physiological function in presynaptic terminals (Burré, 2015). Although LB are characterized by β-sheet aggregated fibrils of α-Syn (Spillantini et al., 1997; Rodriguez et al., 2015), *in vitro* studies have shown that the formation of oligomers, not fibrills, are a distinct hallmark of α-Syn mutations such as A30P and A53T, which are known to cause early-onset familial PD (Conway et al., 2000). The NAC domain of α-Syn is a hydrophobic sequence formed of 12 amino acids which forms the core part of the α-Syn protein and is key for its transformation from soluble monomer to oligomers, into protofibrils and finally aggregating fibrils (Figure 2B; Giasson et al., 2001). *In vitro* studies performed by Narhi and colleagues demonstrated that wild type, as well as mutant A30P and A53T α-Syn can form insoluble β-sheet structured fibrillar aggregates at physiological temperature (Narhi et al., 1999). Importantly, mutant forms of α-Syn promote fibril formation more rapidly than the wild type α-Syn protein. The carriers of A30P and A53T α-Syn point mutations are also more prone to α-Syn aggregation and toxicity, hence correlating with increased vulnerability and earlier onset of PD formation in *SCNA* mutant carriers (Li et al., 2001; Ingelsson, 2016).

Aggregated and oligomeric α-Syn are also implicated in prion-like transmission of progressive PD pathology along distant but connected brain regions (Spillantini et al., 1997; Jucker and Walker, 2013). The accumulation of α-Syn in LB undergo an ascending pattern of distribution, from the lower brainstem and olfactory bulb into the limbic system and ultimately to the neocortex (Braak et al., 2003). Several hypotheses attempt to address the cellular mechanisms underlying progressive α-Syn transmission spreading throughout these brain regions (Longhena et al., 2017). Few studies have supported that cell-to-cell transmissibility of α-Syn occurs at the synapses, through physiological processes such as exocytosis and endocytosis or due to synaptic degeneration (Frost and Diamond, 2009; Guo and Lee, 2014). Although the underlying mechanisms so far remained elusive, there is strong evidence that progressive transmission correlates with severity of the disease (Hawkes et al., 2010) (Box 1).

## Where does PD pathology start?

**Figure 3:**
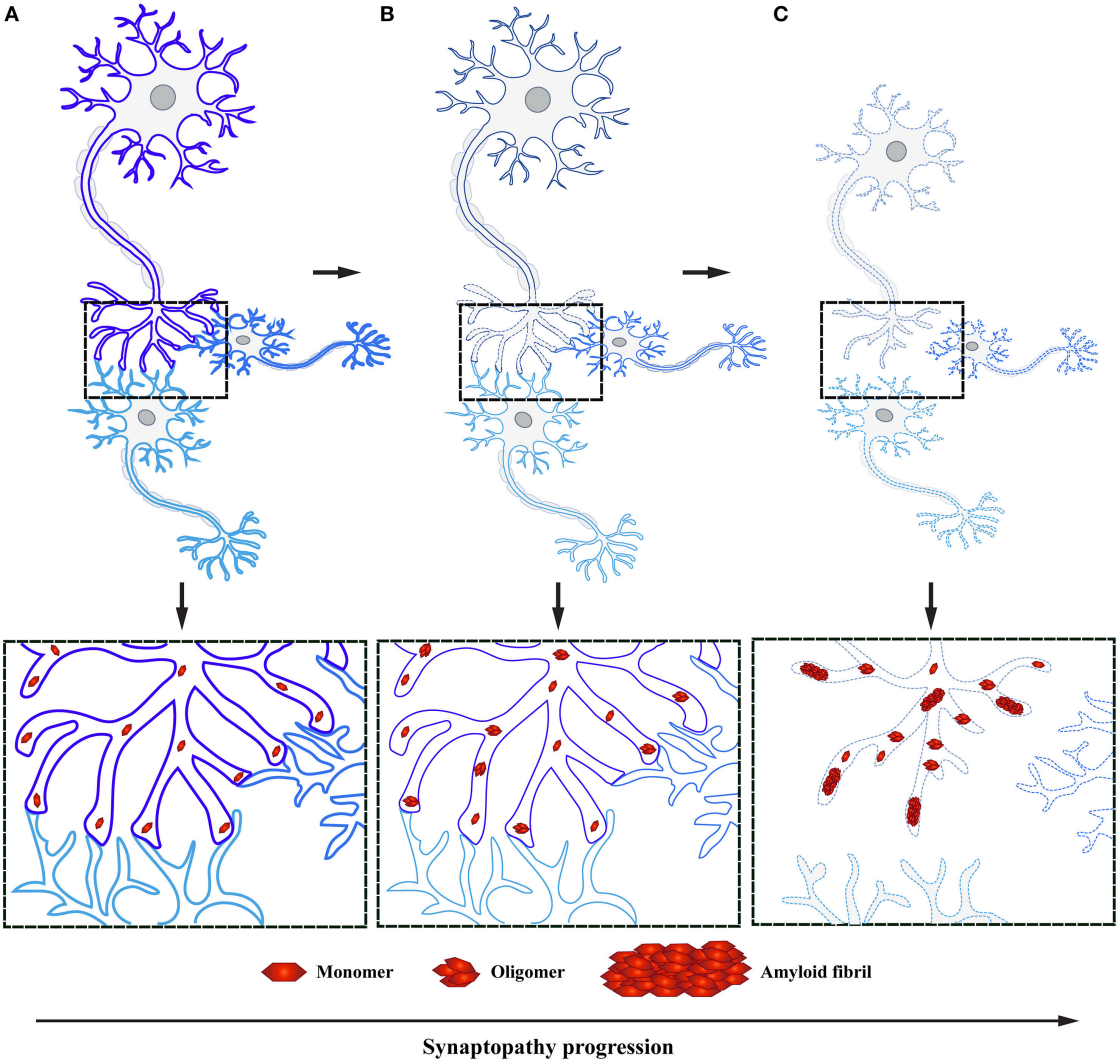
α-Syn accumulation in presynaptic terminals causes synaptopathy ultimately leading to dying back-like neurodegeneration. **(A)** Under physiological conditions, α-Syn functions as monomers at the presynaptic terminals in synaptic transmission. **(B)** Formation of toxic α-Syn species, such as oligomers and fibrils, have been shown to play a pivotal role in PD pathogenesis. These toxic species accumulate at the presynaptic terminal, leading to altered levels of proteins involved in synaptic transmission, ultimately causing synaptic dysfunction. **(C)** As a result of toxic α-Syn accumulation, affected synapses will undergo a process of active deconstruction leading to loss of neuronal connections and subsequent death of the neuronal perikarya.

Several neurodegenerative diseases exhibit early impairment of synaptic function (Bae and Kim, 2017). This often occurs concomitantly with the manifestation of cognitive symptoms, with a neuronal degeneration emerging at later stages of disease (Milnerwood and Raymond, 2010; Schulz-Schaeffer, 2010; Picconi et al., 2012). Thus, synaptic dysfunction is considered to be the first step followed by active deconstruction of axons and loss of neuronal connectivity, eventually leading to the death of neuronal perikarya (Figure 3; Scott et al., 2010; Lu et al., 2014; Morales et al., 2015; Schulz-Schaeffer, 2015; Calo et al., 2016; Grosch et al., 2016; Tagliaferro and Burke, 2016; Bae and Kim, 2017; Fang et al., 2017; Kouroupi et al., 2017; Roy, 2017). This succession of events suggests that neural death in PD is initiated at synaptic terminals and progresses proximally toward neural cell bodies in a dying back-like manner.

In line with this hypothesis, almost 20 years ago Hornykiewicz suggested that formation of PD starts by affecting axons in the dorsal striatum prior to degeneration of DA neurons in the SN (Hornykiewicz, 1998). However, this hypothesis only recently gained strong support by a range of pathological and molecular data. The first evidence that axons were affected in PD came from the pioneer study performed by Braak et al. (1999). They were able to demonstrate that extensive and very thin α-Syn inclusions were not only present in LB inclusions at the neuron soma but also present in axonal processes (Braak et al., 1999). More recent studies found α-Syn localized within axonal dystrophic neurites in the striatum of patients with Alzheimer's disease (AD) and PD (Duda et al., 2002), as well as in multiple system atrophy (MSA) and dementia with Lewy body (DLB) (Galvin et al., 1999). The localization of α-Syn was further studied through the development of a technique called paraffin-embedded tissue (Kramer and Schulz-Schaeffer, 2007). This method allowed for the detection of abundant α-Syn micro-aggregates in the neuropil rather than in the cell body of DLB patient brains. Furthermore, Kramer and Schulz-Schaeffer showed the abundance of synaptic α-Syn micro-aggregates exceeded the amount of α-Syn aggregates in LB or LN by one to two orders of magnitude. Their filtration technique revealed 50–92% of α-Syn micro-aggregates were found entrapped within the presynaptic terminal. This result correlated with the striking downregulation of presynaptic proteins like syntaxin and synaptophysin, and postsynaptic proteins such as PSD95 and drebrin (Kramer and Schulz-Schaeffer, 2007).

This new mechanistic insight exploring synaptic deficits as a starting point in α-Syn related pathology is in agreement with previous findings, where α-Syn aggregates were observed in axon terminals preceding the formation of LB in DLB and correlating with cognitive impairment (Marui et al., 2002). Moreover, similar to DLB, PD patient data demonstrates that these individuals experience first locomotor symptoms once 50–60% of DA striatal terminals have already been lost, while the loss of DA neurons in the SN is believed to be only around 30% (reviewed in Burke and O'Malley, 2013). These observations have been independently confirmed by positron emission tomography; PD patients in early stages of the disease show extensive axonal damage and loss of nigrostriatal pathway connectivity (Caminiti et al., 2017). This data suggests that α-Syn pathology is abundant in presynaptic terminals and axons, in line with the observation that its normal localization is predominantly in presynaptic terminals (Figure 3). Yet little is known how early accumulation of toxic α-Syn species impairs synaptic homeostasis and function, ultimately leading to DA neurodegeneration in PD.

## The Janus face of presynaptic α-Syn: physiology and pathology

**Figure 4:**
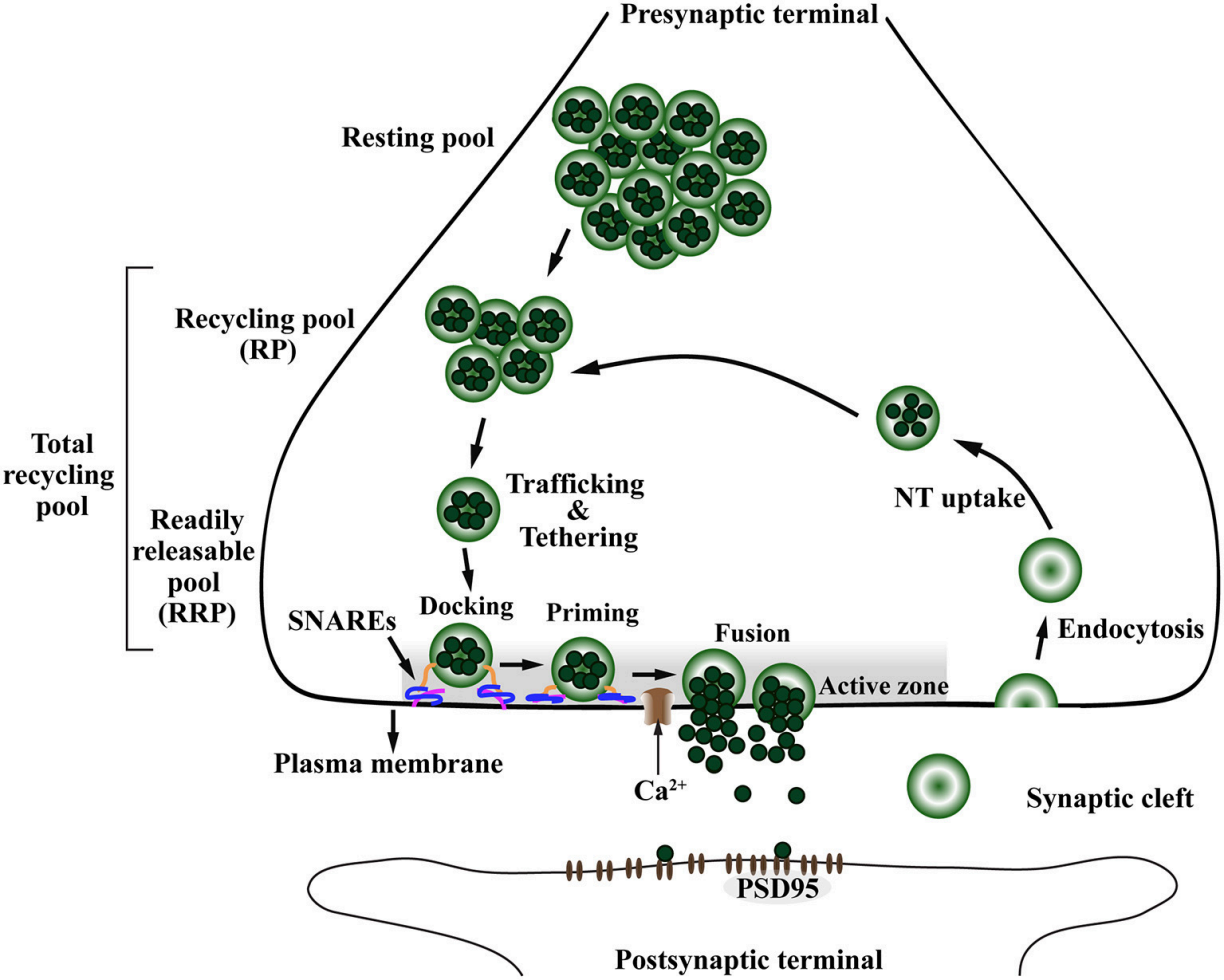
The presynaptic exo-endocytotic cycle regulating neurotransmitter release is specifically affected in α-Syn-related synaptopathies. Upon an incoming action potential, calcium (Ca^2+^) channels become permeable to Ca^2+^ entry in the presynaptic terminal. This activates a molecular machinery including the SNARE complex proteins that recruit synaptic vesicles (SV) from the proximal resting and recycling (RP) pools via trafficking and tethering to form the readily releasable pool (RRP). After docking and priming, RRP vesicles undergo SNARE-mediated membrane fusion at the active zone (AZ, shaded area), ultimately leading to neurotransmitter (NT) release into the synaptic cleft. After exocytosis, the SV membrane is retrieved to the presynaptic terminal via endocytosis, to be filled with NT (NT uptake) and re-enter the exo-endocytotic cycle, thereby granting the neuron its ability to sustain high firing rates. PSD95, postsynaptic density protein-95.

Synapses are the intercellular junctions between a presynaptic neuron and a postsynaptic cell (Südhof, 2012). They are used to transmit signal between neurons in the CNS via exocytosis of neurotransmitter allowing an organized flux of information through the brain (Lepeta et al., 2016). Synapses are composed of a presynaptic terminal, synaptic cleft and postsynaptic terminal which require a complex and tight regulation for proper neurotransmission to occur (Figure 4; Südhof and Rizo, 2011; Südhof, 2012).

The presynaptic nerve terminal is a specialized secretory machinery that releases neurotransmitters by synaptic vesicle (SV) exocytosis in response to an action potential. It is at the presynaptic terminal where α-Syn is hypothesized to play its physiological or pathological role (Katz, 1969; Chandra et al., 2005; Burré, 2015). By receiving an action potential, calcium (Ca^2+^) channels open, thereby allowing influx of Ca^2+^ into the presynaptic terminal (Südhof, 2013b). This Ca^2+^ influx promotes the assembly of a molecular machinery composed mainly of the Ca^2+^ sensor synaptotagmin and the soluble NSF-attachment protein receptor (SNARE) complex, including VAMP-2, SNAP-25, and Syntaxin-1 (Südhof, 2013a). Ultimately, this molecular machinery will trigger SV trafficking from the closest pool, the readily releasable pool (RRP), to be tethered, docked and fused to the presynaptic plasma membrane at a defined region of the presynaptic terminal called the active zone (AZ) (Figure 4), for subsequent release of neurotransmitter in the synaptic cleft (Südhof, 2000, 2012, 2013b; Alabi and Tsien, 2012). In addition to the SNARE complex, a subfamily of highly conserved small GTPases called Rab are implicated in intracellular trafficking of vesicles and the recruitment of SV in the AZ (Binotti et al., 2016).

The AZ matrix is composed of several proteins including RIM, ELKS (also known as ERC/CAST/Rab6IP2), Munc13, Bassoon/Piccolo, Rim-BP, and Liprin-α (Schoch and Gundelfinger, 2006; Südhof, 2012; Wang et al., 2016). The AZ is a highly organized structure restricted to the plasma membrane region, opposite to the postsynaptic density marked by postsynaptic density protein 95 (PSD95). Functionally, AZ proteins dock SVs to be fused with the plasma membrane and release their neurotransmitter content into the synaptic cleft (Figure 4; Wang et al., 2016). After exocytosis, the SV membrane is retrieved from the synaptic cleft by endocytosis, either via clathrin-dependent slow or clathrin-independent fast modes of endocytosis (Saheki and De Camilli, 2012). The SV are locally loaded with neurotransmitter, hence being recycled to allow another round of exo-endocytotic membrane cycling (Südhof, 2004; Gross and von Gersdorff, 2016). This mechanism allows the neurons to sustain a high firing rate without depletion of the SV pools (Saheki and De Camilli, 2012; Gross and von Gersdorff, 2016).

Deficiencies in synaptic transmission have been observed in response to both knock down or overexpression of α-Syn, suggesting that it has a role in the regulation of neurotransmitter release, synaptic function and homeostasis (Box 1) (Kahle et al., 2000; Lee et al., 2008; Zhang et al., 2008; Lashuel et al., 2013; Vargas et al., 2017). Under physiological conditions, α-Syn is found at the presynaptic terminal (Maroteaux et al., 1988) and exists in a dynamic equilibrium between a soluble and a membrane-bound state (Burré, 2015). Specifically, α-Syn is localized to presynaptic boutons due to its preference for membranes with high curvature (Middleton and Rhoades, 2010; Jensen et al., 2011). Despite its known presynaptic localization, the physiological functions of α-Syn remain poorly understood. In the following sections, we will discuss the evidence for physiological and pathological roles of α-Syn in the presynaptic terminal.

### α-Syn affects synaptic vesicle pools and their trafficking in the presynaptic terminal

The SV pools can be divided accordingly to the number of SVs and distance from the AZ (Südhof, 2000; Alabi and Tsien, 2012). The RRP is the closest in proximity to the AZ, containing the smallest number of vesicles and holding the highest fusion probability. The recycling pool (RP) is located above the RRP and has three times more vesicles than the RRP. The RP repopulates vacancies at the RRP which are often rate-limiting during persistent activity and along with the RRP compose the total recycling pool (Alabi and Tsien, 2012). Finally, the most distal SV pool is called the resting pool, this is the largest pool of SVs that remain unreleased even after prolonged synaptic stimulation (Figure 4; Südhof, 2000; Alabi and Tsien, 2012).

α-Syn has been shown to be associated with the recycling pool of SVs and its proteins. Depletion of α-Syn, through the use of antisense oligonucleotides, induces a decrease in the availability of reserve synaptic vesicle pool in primary cultured hippocampal neurons (Murphy et al., 2000). α-Syn knockout (KO) mice revealed a striking deficiency of undocked vesicles without affecting docked vesicles at the RRP (Cabin et al., 2002). In addition, the refilling of the docked vesicles by the resting pool after depletion was slower in the synapses of mice lacking α-Syn (Cabin et al., 2002). Together this data strongly supports a role of α-Syn in SV trafficking at the presynaptic terminal by modulating vesicular dynamics and maintaining the homeostasis of vesicle RPs.

Overexpression of wild type and mutant α-Syn in Chromaffin and PC12 cells revealed inhibited evoked neurotransmitter release (Larsen et al., 2006). This inhibition was attributed to a smaller population of RRP, suggesting that α-Syn inhibits the vesicle “priming” step which is the intermediate step between vesicle docking and fusion of synaptic vesicles at the AZ (Larsen et al., 2006). Furthermore, overexpression of wild type α-Syn to similar levels observed in PD patients with duplication and triplication of the *SNCA* gene, inhibited neurotransmitter exocytosis by affecting the size of the RP (Nemani et al., 2010; Scott and Roy, 2012).

Interestingly, ultrastructural analysis of the synapse in mice overexpressing human wild type α-Syn demonstrated the total number of SVs in the presynaptic terminal was not altered. Instead, these animals displayed reduced numbers of SVs at a close proximity to the AZ, whereas numbers of SVs at greater distances were increased (Nemani et al., 2010). Furthermore, this study concluded that exocytotic deficits caused by α-Syn overexpression were not only caused by reduced size of the RP, but also due to disturbances of SV reclustering after endocytosis (Nemani et al., 2010). These findings demonstrate high levels of α-Syn can trigger impairment of exocytosis via alteration of SV pools in absence of neurodegeneration.

### The SNARE complex related to α-Syn function and dysfunction

The SNARE proteins are crucial to allow SVs to fuse with the plasma membrane at the AZ to release neurotransmitter (Südhof and Rizo, 2011). The subsequent exocytosis requires the assembly of presynaptic machinery proteins thousands of times per minute. During each episode, the SNARE complex assembly and disassembly gives rise to extremely reactive unfolded SNARE protein intermediates, exposing the presynaptic terminal to potentially vulnerable activity-dependent degeneration (Burré et al., 2010; Südhof and Rizo, 2011). α-Syn has been shown to act as a chaperone to promote SNARE complex assembly and to support its folding through direct binding to VAMP-2 and phospholipids on synaptic vesicles (Burré et al., 2010).

**Figure 5:**
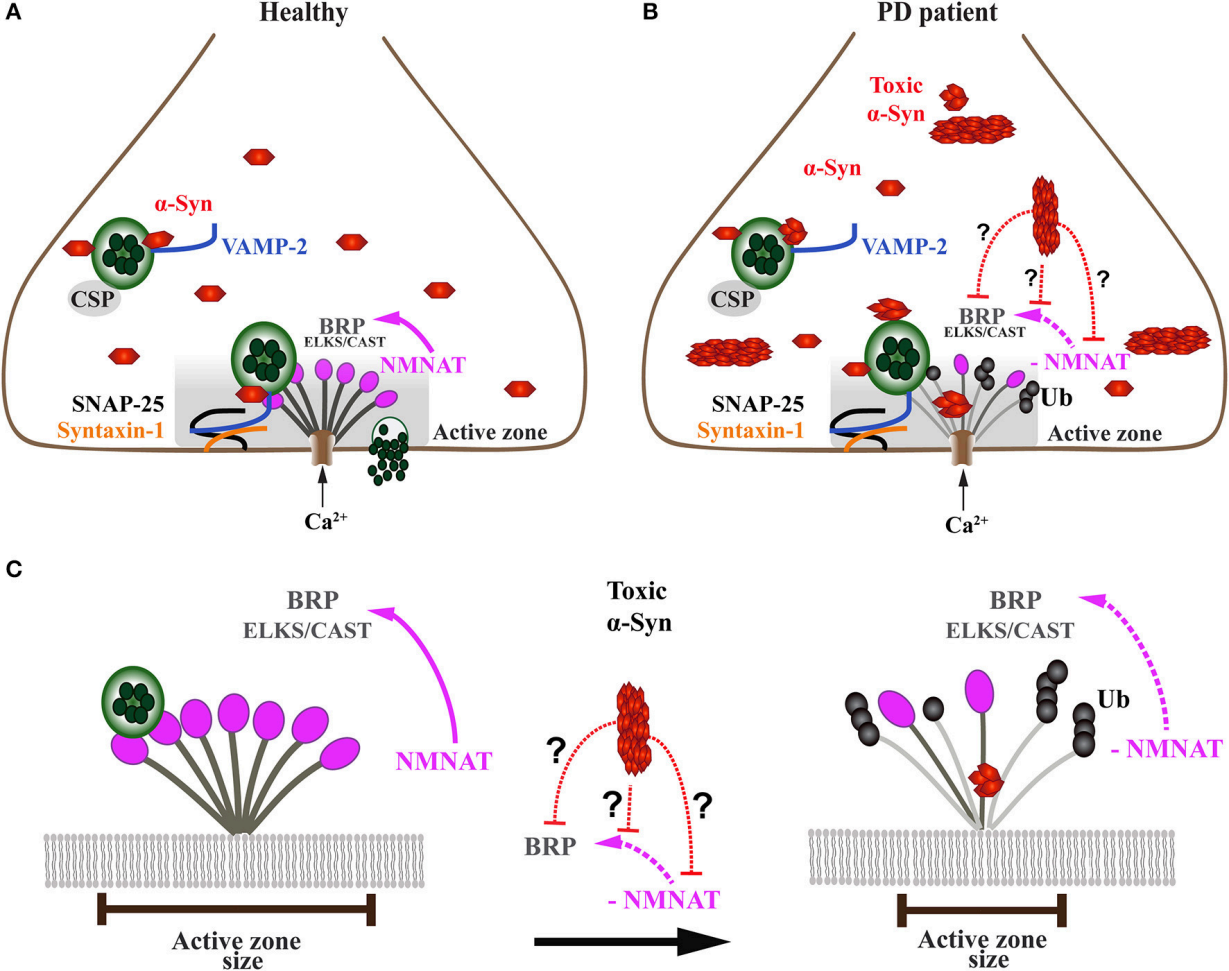
Onset of α-Syn-mediated synaptopathies is likely related to impaired presynaptic active zone function and defective neurotransmitter release. **(A)** In healthy subjects, the presynaptic terminal of a neuron comprises a functional exo-endocytotic cycling machinery, including vesicle pools, SNARE complex proteins and the active zone (AZ) which is formed of a dense network of proteins called AZ matrix. The AZ matrix is the site of action for synaptic vesicle tethering, docking and membrane fusion for neurotransmitter release which is mediated by SNARE complex proteins including VAMP-2, SNAP-25, and Syntaxin-1. This process is directed and co-chaperoned by CSP and α-Syn toward the AZ, a scaffold that includes ELKS/CAST proteins, the *Drosophila* homolog of Bruchpilot (BRP). BRP mutants have been shown to cause dramatic reduction of nerve-evoked transmission. Maintenance of BRP within the AZ matrix scaffold is dependent on direct interaction with NMNAT which protects BRP against ubiquitin (Ub)-mediated degradation and activity-induced neurodegeneration. **(B)** In PD patients, presynaptic neuronal terminals accumulate toxic oligomers and protofibrils of α-Syn that interfere with the exo-endocytotic cycling machinery, including AZ proteins NMNAT and BRP/ELKS/CAST (enlarged in **C**). **(C)** Disease-related downregulation of NMNAT leads to ubiquitination and degradation of BRP/ELKS/CAST, resulting in the gradual decrease and eventual dissolution of the AZ matrix, ultimately causing impaired synaptic transmission and α-Syn-mediated synaptopathy. However, it is currently unknown (doted red lines) whether toxic forms of α-Syn directly or indirectly interfere with NMNAT and/or BRP/ELKS/CAST, thereby causing the AZ to lose its physiological function.

In line with these observations, α-Syn overexpression is able to rescue neurodegeneration observed in CSPα KO mice by chaperoning the assembly of the SNARE complex, while concomitant knockout of α-Syn exacerbates the CSPα KO phenotype (Chandra et al., 2005). CSPα acts as a chaperone of the SNARE complex by supporting the functional ability of SNAP-25 to engage in a complex with Syntaxin-1 and VAMP-2 (Figure 5A; Sharma et al., 2011). The chaperone function of CSPα within the SNARE complex is shared with α-Syn, however the mechanisms differ. α-Syn binds to VAMP-2 via its C-terminal (Burré et al., 2010) which appears to be essential for α-Syn mediated neuroprotection because the A30P α-Syn mutant, which is deficient in its ability to bind to membranes, failed to rescue CSPα KO mice (Chandra et al., 2005). This role of native α-Syn in assisting SNARE complex assembly contributes to clustering of SV at the AZ (Figure 5A). Besides functioning as chaperone, α-Syn is also important for maintenance and redistribution of the SNARE complex which is directly implicated in neurotransmitter release, including dopamine (Burré et al., 2010; Diao et al., 2013).

The synaptic accumulation of oligomers and aggregated α-Syn and its interaction with components of the presynaptic machinery, such as SNARE proteins, is likely to underlie at least one of the pathological mechanisms that lead to synaptic dysfunction in the early stages of PD (Box 1) (Figure 5B). In support of this hypothesis, studies *in vitro* have demonstrated large α-Syn oligomers bind to the N-terminal domain of VAMP-2, thus blocking SNARE-mediated vesicle docking (Choi et al., 2013), which *in vivo* could be directly linked to impaired exocytosis of neurotransmitters in overexpression models of α-Syn. Interestingly, another *in vitro* study by Lai and co-authors revealed that levels and different toxic species of α-Syn inhibit synaptic vesicle docking via distinct mechanisms (Lai et al., 2014). These data suggest that α-Syn may form various types of oligomeric states with different toxicity, as has been shown for aggregated tau protein in tauopathies (Goedert, 2015). Moreover, Scott and colleagues showed that cultured hippocampal neurons from transgenic animals overexpressing human α-Syn displayed absence of endogenous synaptic proteins, including the SNARE complex protein VAMP-2 and SV proteins amphiphysin and synapsin-1 (Scott et al., 2010). Neurons overexpressing fluorescent human α-Syn demonstrated marked deficits in neurotransmitter release, in addition to reduced numbers of SVs, and enlargement of the remaining SVs (Scott et al., 2010). Studies investigating levels of synaptic proteins belonging to the SNARE complex in PD, PD with dementia (PDD) and DLB post-mortem samples also reported misregulation of proteins belonging to the SNARE complex (Kramer and Schulz-Schaeffer, 2007; Dijkstra et al., 2015; Bereczki et al., 2016, 2018). Moreover, an *in vitro* model of α-Syn toxicity, primary neuronal cultures exposed to preformed α-Syn fibrils exhibited loss of the SNARE complex proteins VAMP2 and SNAP-25, as well as the SV proteins CSPα and synapsin-2 (Volpicelli-Daley et al., 2011). Volpicelli-Daley and colleagues further investigated the impact of accumulation of these preformed α-Syn fibrils in neural network activity. Interestingly, they found that impaired hippocampal network activity occurred much earlier than the detected reduction in synaptic protein levels, suggesting that α-Syn pathology can have a major impact on the coordination of neuronal communication and connectivity (Box 1) (Volpicelli-Daley et al., 2011).

Conversely, a mouse model expressing human truncated α-Syn leading to α-Syn aggregates did not show changes in levels of synaptic proteins but rather caused age-dependent redistribution and accumulation of SNAP-25, Syntaxin-1, and VAMP-2 in the striatum accompanied by an age-dependent reduction in dopamine release (Garcia-Reitböck et al., 2010). SNARE proteins were also redistributed in the striatum and external globus pallidus of early-onset PD patients (Garcia-Reitböck et al., 2010). Taken together, evidence from transgenic mouse models and PD patients demonstrate that accumulated α-Syn alters the levels and/or localization of SNARE proteins at the presynaptic nerve terminal. These findings suggest that α-Syn accumulation causes a toxic gain of function phenotype at the synapses, thus impairing their function and connectivity.

### Accumulated α-Syn impairs rabs and their function in synaptic vesicle trafficking

The trafficking of synaptic vesicles within the presynaptic terminal is vital to recruit them from the SV pools into the active zone (Figure 4; Südhof, 2000). This is a complex process that involves a significant number of proteins and must be tightly controlled (Takamori et al., 2006; Südhof, 2013b). Amongst the proteins involved in presynaptic vesicle trafficking, a superfamily of highly conserved small GTPases called Rab act as the master regulator of intracellular trafficking processes, including vesicle budding and uncoating, motility, tethering and fusion (reviewed in Kelly et al., 2012). Rab3A protein, for instance, is abundant in SV with its activity regulated by two effectors: Rabphilin and RIM (for Rab3 interacting molecules; Shirataki et al., 1993; Wang et al., 1997). Mechanistically, Rab3A is part of a complex formed by the AZ proteins Munc13 and RIM, designed to capture SVs for final membrane attachment called tethering and to prepare them for synaptic release (Wang et al., 1997; Dulubova et al., 2005).

α-Syn was shown to interact and cause defects in vesicular trafficking pathways including those taking place at the presynaptic terminal of neurons (Dalfó et al., 2004; Gitler et al., 2008; Abeliovich and Gitler, 2016). In line with this observation, several members of Rab GTPase family have been identified to interplay with α-Syn (Chutna et al., 2014; Gonçalves et al., 2016). This interaction has also been observed in post-mortem tissue of DLB and MSA patients with LB pathology where α-Syn abnormally interacts with Rab3A. In addition, α-Syn aggregates were found to trap Rab3A and prevent its interaction with rabphilin, thus suggesting that exocytosis and neurotransmitter release are affected in Lewy body diseases (Dalfó et al., 2004). Further studies have proposed that Rab3A regulates membrane association of α-Syn in a GTP-dependent manner (Chen et al., 2013), implying functional integration of α-Syn into Rab proteins cycle between the cytosol and the membrane-bound state, and hence into the SV cycle (Bendor et al., 2013).

These functional interactions are further supported in a *Caenorhabditis elegans* model overexpressing wild type human α-Syn which revealed that manipulating levels of Rab3A, Rab1 and Rab8A significantly rescued α-Syn mediated loss of DA neurons (Gitler et al., 2008). Similar effects were seen in rat midbrain cultures transfected with A53T mutant α-Syn (Gitler et al., 2008). α-Syn mediated neurotoxicity was hypothesized to be caused by α-Syn affecting transport of SV via direct interaction with the trafficking machinery (Gitler et al., 2008). Furthermore, studies in *Drosophila melanogaster* suggest that Rab3 is also involved in the formation of the AZ architecture, however the mechanisms remain unknown (Chen et al., 2015; Bae et al., 2016).

### The active zone matrix and α-Syn

The docking and fusion of SVs are restricted to the active zone, demonstrating that the AZ is essential to translate an incoming action potential into neurotransmitter release (Figure 4; Schoch and Gundelfinger, 2006; Südhof, 2012; Wang et al., 2016). The AZ is a highly conserved specialized presynaptic scaffold characterized by a dense network of proteins called AZ matrix. In mammals, this matrix includes RIM, ELKS, Munc13, Bassoon/Piccolo, Rim-BP, and Liprin-α (Schoch and Gundelfinger, 2006; Südhof, 2012; Wang et al., 2016). Furthermore, additional proteins are present but not restricted to the AZ, including SNAREs, ion channels, receptors, cytoskeletal proteins and adhesion molecules (Wang et al., 2016). Considering the high number of proteins constituting the AZ, it is important for this presynaptic compartment to be tightly regulated according to synaptic activity for reliable neurotransmitter release to occur (Körber and Kuner, 2016).

Although a large amount of evidence indicates that accumulation of α-Syn at the presynaptic terminal causes reduction of proteins involved in synaptic transmission, to our knowledge, α-Syn has not been shown to directly interact with AZ matrix proteins under normal conditions. In addition to that, only a few studies have explored the pathological role of α-Syn affecting proteins associated with the AZ. Mantin and colleagues reported mice overexpressing human mutant A53T α-Syn which showed abnormal inclusions of α-Syn within dendrites and in the presynaptic AZ (Martin et al., 2006). Furthermore, a recent ultrastructural study by Kohl and colleagues utilized a bacterial artificial chromosome (BAC) rat model expressing full-length human *SNCA* gene. These transgenic rats displayed extended AZs in synapses of hippocampal CA3 neurons compared to non-transgenic animals, a phenotype that was accompanied by reduction of the postsynaptic density. However, PSD95 levels were found to be upregulated in the dentate gyrus/CA3 regions in combination with downregulation of synapsin1 and RIM3 (Kohl et al., 2016). This study suggests that α-Syn does not only compromise the presynaptic but also postsynaptic terminals by altering both RIM3 and PSD95. The RIM protein family is an essential regulator of active zone function (Mittelstaedt et al., 2010; Südhof and Rizo, 2011). Another AZ protein called Piccolo was shown to be absent in a significant number of synaptic boutons in a model overexpressing α-Syn (Scott et al., 2010). Piccolo along with Bassoon are specific to the vertebrate AZ and do appear to have a major function in guiding SVs to the AZ (Mukherjee et al., 2010; Südhof, 2012).

Since α-Syn has been demonstrated to accumulate at the AZ (Martin et al., 2006), it is reasonable to hypothesize that AZ matrix proteins become dysfunctional over time. Moreover, α-Syn accumulation in the AZ may jeopardize a compensatory activity of AZ proteins to overcome the upstream deleterious effects caused by toxic α-Syn, such as alterations of SV pools, SNAREs, and intracellular trafficking that together lead to impaired neurotransmitter exocytosis (Larsen et al., 2006; Nemani et al., 2010; Scott et al., 2010; Janezic et al., 2013). Furthermore, the accumulation of toxic α-Syn species in the AZ triggers the proteasome system to degrade not only α-Syn but also AZ matrix components, the levels of which are normally regulated by proteasome activity (Wang et al., 2017).

Work carried out in *Drosophila melanogaster* has provided at least one mechanistic pathway by which the presynaptic AZ is sustained under disease-related stress (Figure 5). The maintenance of the AZ structure against activity-induced degeneration is provided by a chaperone called *Drosophila* nicotinamide mononucleotide adenylyltransferase (dNMNAT) (Zang et al., 2013). This neuroprotective effect is provided through NMNAT, which protects the AZ protein Bruchpilot (BRP), a homolog of mammalian ELKS/CAST proteins, from ubiquitination and subsequent degradation. However, it is not known whether high levels and/or toxic α-Syn species, such as oligomers and fibrils, exert a toxic gain of function onto the AZ by interfering with NMNAT function (Figures 5B,C).

Unlike *Drosophila* that only encodes one NMNAT homolog, mammals possess three different NMNAT orthologues that are distinguishable by their differential localization within subcellular compartments (Brazill et al., 2017). NMNAT1 is a nuclear protein, NMNAT2 is localized to the Golgi apparatus and NMNAT 3 is predominantly mitochondrial (Ali et al., 2013). NMNATs are classically known for their enzymatic function of catalyzing NAD+ synthesis, which is an essential cofactor in many cellular processes including oxidative reactions and transcriptional regulation (Lau et al., 2009). NMNAT is also able to act as a molecular chaperone (reviewed in Ali et al., 2013), which is critical for neuronal homeostasis and protection against cellular stress (Ali et al., 2012; Ocampo et al., 2013; Rallis et al., 2013). However, this role does not require the enzymatic function of NMNAT, as the inactive form of dNMNAT was able to protect *Drosophila* photoreceptors against activity-induced degeneration (Zhai et al., 2006, 2008).

Consistent with the role of NMNAT as a neuronal maintenance factor, it has been shown that NNMAT2, which is enriched in synaptic terminals and SVs, is downregulated in several degenerative disorders. These include PD, AD, Huntington's disease (HD) and frontotemporal lobar degeneration with ubiquitin- and TDP-43-positive inclusions (FTLD-TDP) (Ali et al., 2013). NMNAT2 alterations were also found in a mouse model of tauopathy expressing mis-sense mutation P301L, found in some frontotemporal dementia and parkinsonism linked to chromosome 17 (FTDP-17) cases (Ljungberg et al., 2012). The neuroprotective effects of mammalian NMNAT2 has been explored in more detail in axonal degeneration, where loss of NMNAT2 is considered to be a physiological stimulus in severed axons and their subsequent Wallerian degeneration (Gilley and Coleman, 2010). In line with this notion, NMNAT2 overexpression was found to delay injury-induced axonal degeneration in zebrafish (Feng et al., 2010) and to protect mice against tauopathy-induced neurodegeneration (Ljungberg et al., 2012).

### Accumulated α-Syn affects synaptic vesicle endocytosis

In order to sustain high rates of synaptic transmission without depleting the supply of SVs, neurons have to rely on efficient endocytic recycling of SV membranes (Figure 4; Saheki and De Camilli, 2012). The synuclein family is required for SV endocytosis (Vargas et al., 2014). Using a triple synuclein KO mouse model, Vargas and colleagues demonstrated that synucleins are required for fast and efficient kinetics of early steps of SV endocytosis, membrane bending and cargo selection (Vargas et al., 2014). Fundamentally, the impaired endocytic process observed in the triple KO mouse could be re-established by individual expression of α-, β-, or ɤ-Syn, demonstrating they are functionally redundant in the endocytic process (Vargas et al., 2014).

Rab5, which has been localized to the early endosome and is implicated in the regulation of neuronal endocytosis in association with Rabaptin-5 (Stenmark et al., 1994; Cataldo et al., 2000), appears to play a role in the internalization of exogenous α-Syn. Overexpression of constitutively active Rab5 or its effector Rabaptin-5 in cultured neurons lead to the formation of intracellular inclusions resembling Lewy bodies (Sung et al., 2001). More recently, a study revealed that high levels of α-Syn promotes its interaction with dynein, a retrograde motor protein, inducing a significant increase in activated levels of Rab5 and Rab7, both key regulators of the endocytic process (Fang et al., 2017).

Under intense electric stimulation, overexpression of wild type α-Syn in lamprey nerve terminals caused loss of SVs and an expanded plasma membrane which pointed toward impaired vesicle recycling (Busch et al., 2014). However, this endocytic deficit was not observed when low stimulation was applied. Interestingly, overexpression of mutant A30P α-Syn, with a disrupted N-terminal that greatly impairs its binding to phospholipids and disrupt the α-helical conformation, showed very little effect on vesicle recycling. Therefore, Busch and colleagues concluded that synaptic endocytic failure could only be visualized when high synaptic activity is in place and under proper folding of α-Syn N-terminal α-helix (Busch et al., 2014). Corroborating these findings, overexpression of mutant human A53T α-Syn, but not A30P α-Syn, at the calyx of Held terminals in mice was also shown to cause impairment of slow and rapid endocytosis, and restoration of the RRP (Xu et al., 2016). These studies endorse the role of α-Syn in membrane curvature formation and stabilization, and consequently for endocytosis (Pranke et al., 2011; Busch et al., 2014).

### α-Syn related presynaptic deficits converge in dopaminergic neurons

Under physiological conditions, native soluble α-Syn is involved in several steps in the presynaptic terminal that are required to trigger exocytosis upon activation by an action potential (Burré, 2015). However, these steps are not exclusively regulated by α-Syn. Under pathological conditions, the presence of toxic α-Syn species such as oligomers and amyloid fibrils, triggers the misregulation of several synaptic proteins (Table 1), leading to functional impairment of the presynaptic terminal in both animal models and PD patients (Figure 5). These alterations at the presynapse have been shown to be progressive and selective to dopaminergic neurons, at least in mouse models (Volpicelli-Daley et al., 2011; Janezic et al., 2013). Thus, upon α-Syn accumulation, DA neurons show reduced firing rates, a phenotype that positively correlates with behavior impairment, ultimately followed by degeneration of these neurons (Janezic et al., 2013).

A similar sequence of events whereby presynaptic deficits precede neuronal loss are also recapitulated in induced pluripotent stem cells (iPSCs) derived from PD patients that harbor the A53T α-Syn mutation. Kouroupi and colleagues observed a significant downregulation of genes coding for presynptic proteins in comparison to controls (Kouroupi et al., 2017). Strikingly, A53T α-Syn iPSCs cells exhibited thinner, less fasciculated neuronal processes that were accompanied by a 27% reduction in the number of synaptic contacts. The deleterious effects caused by overexpression of human α-Syn were also observed in *Drosophila* which identified reduced synaptic connectivity between DA neurons and Kenyon cells even before the onset of motor abnormalities (Riemensperger et al., 2013).

Together this data suggests that there is a potential direct association, between α-Syn accumulation and dopamine metabolism, which in turn makes DA neurons especially vulnerable to cellular stress and subsequent neurodegeneration. Indeed, several recent findings support this hypothesis, which could shed light onto the pathogenic mechanisms underlying DA neuron-specific cell death in PD.

## α-Syn and dopamine: a dangerous liaison

The loss of dopaminergic neurons in the substantia nigra along with α-Syn enriched Lewy body inclusions are the main histopathological hallmarks of PD (Lees et al., 2009). DA neurons are thought to be the most vulnerable cell type in PD, which led to several hypotheses attempting to explain the underlying mechanisms. One of these is related to the high-energy requirement of DA neurons due to their poorly myelinated axonal arborisation which is orders of magnitude larger than any other neuronal cell type (Pissadaki and Bolam, 2013). This hypothesis predicts that any disturbance inducing a negative energy balance would be especially dangerous to DA neurons, placing them at high risk in case of energy failure, such as mitochondrial dysfunction, which has been shown to play an important role in PD (Lin and Beal, 2006).

Although energy balance contributes to the vulnerability of DA neurons, regulation of dopamine levels at the synaptic terminals is thought to be vital for DA neuron susceptibility observed in PD (Lotharius and Brundin, 2002b). Accordingly, α-Syn has been implicated as a key regulator of dopamine homeostasis (Abeliovich et al., 2000; Venda et al., 2010). The activity of the rate-limiting enzyme tyrosine hydroxylase (TH) responsible for producing dopamine through conversion of L-tyrosine to L-3,4-dihydroxyphenylalanine (L-DOPA), is negatively regulated by increased concentration of α-Syn *in vitro* (Perez et al., 2002). As a result, reduced levels of dopamine were observed in cell culture transfected with A53T mutant α-Syn (Perez et al., 2002).

In line with these findings, reduced TH activity was found in mouse models overexpressing α-Syn (Masliah et al., 2000; Kirik et al., 2002). Mechanistically, α-Syn decreases TH activity by reducing its phosphorylation *in vitro* (Perez et al., 2002; Peng et al., 2005) as TH is activated by phosphorylation of seryl residues in the TH regulatory domain (Alerte et al., 2008). In turn, by silencing α-Syn in MN9D cells, increased TH activity and dopamine biosynthesis were observed (Liu et al., 2008). In addition to the ability of α-Syn to regulate TH activity, work performed by Tehranian and colleagues demonstrated that α-Syn can interact with L-aromatic amino acid decarboxylase (AADC) and can reduce its activity (Tehranian et al., 2006). AADC acts in the last step of dopamine synthesis by converting L-DOPA to dopamine (Tehranian et al., 2006).

α-Syn does not only modulate the activity of enzymes responsible for dopamine synthesis, but it also impairs transport and uptake of dopamine by altering the activity of the vesicular monoamine transporter 2 (VMAT2) and the dopamine transporter (DAT), respectively. VMAT2 is responsible for dopamine uptake from the cytoplasm into SV (Lotharius and Brundin, 2002b). DAT transports dopamine from the synaptic cleft back into the cytoplasm is the primary mechanism and possibly the most important regulator of extracellular dopamine concentrations (Vaughan and Foster, 2013). Lundblad and colleagues showed that unilateral overexpression of human wild type α-Syn in the SN of adult rats caused 50% reduction in dopamine reuptake without axonal damage 10 days after exposure (Lundblad et al., 2012). Over time, dopamine reuptake was further decreased in the striatum, followed by axonal swelling and dystrophic axons in the presence of α-Syn aggregates and a profound 70% reduction in dopamine release upon chemical pulse with potassium chloride (Lundblad et al., 2012). Additionally, α-Syn was shown to interact with DAT and in turn to form complexes via the carboxyl-terminal tail of DAT and the NAC domain of α-Syn (Lee et al., 2001). This interaction and complex formation reduced DAT activity due to impairment of dopamine reuptake from the synaptic cleft (Wersinger and Sidhu, 2003; Wersinger et al., 2003), suggesting that α-Syn accumulation causes a vicious cycle of impaired VMAT2 and DAT function.

Consistent with this notion, Bellucci et al. showed that mice expressing the truncated α-syn120 exhibited marked redistribution of DAT/α-Syn complexes in the striatum and the SN (Bellucci et al., 2011). These findings suggest that α-Syn acts as a synaptic modulator of DA in early PD pathogenesis by regulating the subcellular distribution of key proteins such as DAT (Bellucci et al., 2011). DAT has also been reported to be absent in the putamen of PD patients (Seeman and Niznik, 1990). Interestingly, VMAT2 was shown to be incorporated into Lewy bodies of SN neurons in PD patients (Yamamoto et al., 2006). Moreover, profound defects in VMAT2 were also observed in synaptic vesicles isolated from post-mortem brain tissue of PD patients, including reduction in vesicular uptake and the binding ability of VMAT2 (Pifl et al., 2014). These findings are in agreement with previous studies showing that increased levels of α-Syn inhibit VMAT2 activity, ultimately increasing cytosolic levels of dopamine (Guo et al., 2008). As a consequence, dysregulation of dopamine transport caused by impaired DAT and VMAT2 activity will lead to altered levels of dopamine (Lotharius and Brundin, 2002b; Nutt et al., 2004). Dopamine by itself is a risk factor for dopaminergic neurodegeneration; it is highly sensitive to spontaneous degradation and to produce quinone as well as other reactive oxygen species until it is sequestered into vesicles (Lotharius and Brundin, 2002a,b; Xu et al., 2002; Caudle et al., 2007).

This data suggests an intricate and potentially direct interplay between α-Syn and dopamine that directly contributes to functional deficits of DA neurons. In support of this, Mor et al. recently demonstrated that dopamine promotes α-Syn oligomerisation *in vivo* and that disrupting the ability of dopamine to stabilize or modify α-Syn oligomers was sufficient to rescue dopamine-mediated toxicity (Mor et al., 2017). These results recapitulate *in vitro* data revealing the ability of dopamine to trigger formation of α-Syn oligomers (Choi et al., 2013). This cooperative toxicity of α-Syn and dopamine was also recapitulated in *C. elegans* (Mor et al., 2017). Furthermore, a corresponding mouse model revealed early mild synaptic defects in the striatum without degeneration of cell bodies; however, by 5 months post-injection, the mice exhibit a severe loss of dopaminergic nerve terminals (−62% VMAT2 and −55% DAT) as compared to only 25% loss of dopaminergic neurons (Mor et al., 2017). These results reinforce the hypothesis that α-Syn mediated early dopamine imbalance at synaptic terminals is the initiating event which in turn triggers synaptic and axonal dysfunction, subsequently leading to degeneration of nigrostriatal pathways and DA neuron-specific cell death.

## Conclusions

Studies in PD-related animal models and post-mortem patient material demonstrate that soluble oligomers and aggregating protofibrils of α-Syn accumulate in synapses and axons prior to the onset of disease symptoms. These accumulating toxic α-Syn species impair synaptic compartments and presynaptic processes required for neuronal communication, including maintenance of SV pools, SV trafficking, tethering, docking, priming and fusion of SV within the plasma membrane at the active zone. The resulting synaptopathy phenotypes manifest prior to dopaminergic neurodegeneration, hence reinforcing the hypothesis that onset and evolution of PD progress in a dying back-like manner, from initial dysfunction at the synapse and impairing axonal connections, to eventual neuronal cell body death. Although α-Syn is expressed throughout the brain and enriched at presynaptic terminals, DA neurons are most susceptible to toxic effects exerted by accumulating and dysfunctional α-Syn. This fatal attraction is likely related to α-Syn regulating dopamine levels in two distinct manners. First, α-Syn is a negative modulator of dopamine synthesis by interplay with the rate-limiting enzyme TH and AADC. Second, α-Syn disrupts VMAT2 and DAT activity and their function in regulating dopamine levels in the synaptic terminal. In turn, dysregulated dopamine levels fuel the formation of soluble α-Syn oligomers that are considered to be the most toxic species. This fatal interplay and its ensuing vicious cycle of dopamine and α-Syn interference is likely the starting point driving synaptic dysfunction and impaired neuronal communication observed in the early, prodromal stages of PD.

## Author contributions

All authors listed have made a substantial, direct and intellectual contribution to the work, and approved it for publication.

### Conflict of interest statement

The authors declare that the research was conducted in the absence of any commercial or financial relationships that could be construed as a potential conflict of interest. The handling Editor declared a shared affiliation, though no other collaboration, with the authors.
